# Assessing tumor angiogenesis using dynamic contrast-enhanced integrated magnetic resonance-positron emission tomography in patients with non-small-cell lung cancer

**DOI:** 10.1186/s12885-021-08064-4

**Published:** 2021-04-01

**Authors:** Yu-Sen Huang, Jenny Ling-Yu Chen, Hsin-Ming Chen, Li-Hao Yeh, Jin-Yuan Shih, Ruoh-Fang Yen, Yeun-Chung Chang

**Affiliations:** 1 Department of Radiology, National Taiwan University College of Medicine, No. 7, Chung-Shan S. Rd., Taipei, 100 Taiwan; 2 Department of Medical Imaging, National Taiwan University Hospital, Taipei, Taiwan; 3 Department of Oncology, National Taiwan University Hospital, Taipei, Taiwan; 4 National Taiwan University Cancer Center, National Taiwan University College of Medicine, Taipei, Taiwan; 5 Department of Internal Medicine National Taiwan University Hospital, Taipei, Taiwan; 6 Department of Nuclear Medicine, National Taiwan University Hospital, Taipei, Taiwan

**Keywords:** Personalized medicine, Radiologic biomarkers, Angiogenesis inhibitors, Survival

## Abstract

**Background:**

Angiogenesis assessment is important for personalized therapeutic intervention in patients with non-small-cell lung cancer (NSCLC). This study investigated whether radiologic parameters obtained by dynamic contrast-enhanced (DCE)-integrated magnetic resonance-positron emission tomography (MR-PET) could be used to quantitatively assess tumor angiogenesis in NSCLC.

**Methods:**

This prospective cohort study included 75 patients with NSCLC who underwent DCE-integrated MR-PET at diagnosis. The following parameters were analyzed: metabolic tumor volume (MTV), maximum standardized uptake value (SUV_max_), reverse reflux rate constant (*k*_ep_), volume transfer constant (*K*^trans^), blood plasma volume fraction (*v*_*p*_), extracellular extravascular volume fraction (*v*_*e*_), apparent diffusion coefficient (ADC), and initial area under the time-to-signal intensity curve at 60 s post enhancement (iAUC_60_). Serum biomarkers of tumor angiogenesis, including vascular endothelial growth factor-A (VEGF-A), angiogenin, and angiopoietin-1, were measured by enzyme-linked immunosorbent assays simultaneously.

**Results:**

Serum VEGF-A (*p* = 0.002), angiogenin (*p* = 0.023), and Ang-1 (*p* <  0.001) concentrations were significantly elevated in NSCLC patients compared with healthy individuals. MR-PET parameters, including MTV, *K*^trans^, and *k*_ep_, showed strong linear correlations (*p* <  0.001) with serum angiogenesis-related biomarkers. Serum VEGF-A concentrations (*p* = 0.004), MTV values (*p* <  0.001), and *k*_ep_ values (*p* = 0.029) were significantly higher in patients with advanced-stage disease (stage III or IV) than in those with early-stage disease (stage I or II). Patients with initial higher values of angiogenesis-related MR-PET parameters, including MTV > 30 cm^3^ (*p* = 0.046), *K*^trans^ > 200 10^− 3^/min (*p* = 0.069), and *k*_ep_ > 900 10^− 3^/min (*p* = 0.048), may have benefited from angiogenesis inhibitor therapy, which thus led to significantly longer overall survival.

**Conclusions:**

The present findings suggest that DCE-integrated MR-PET provides a reliable, non-invasive, quantitative assessment of tumor angiogenesis; can guide the use of angiogenesis inhibitors toward longer survival; and will play an important role in the personalized treatment of NSCLC.

## Background

Non-small-cell lung cancer (NSCLC) is characterized by poor prognosis and is the leading cause of cancer-related mortality worldwide, and tumor angiogenesis pathways are essential in the process of primary tumor growth, proliferation, and development of distant metastases. Therefore, targeted therapy against angiogenesis has been identified as an important strategy and has now been clinically approved for the first-line treatment of NSCLC in selected patients [1, 2].

Tumor angiogenesis in NSCLC can be non-invasively identified by imaging techniques [3]. Dynamic contrast-enhanced (DCE)-integrated magnetic resonance imaging (MRI) uses permeability and perfusion parameters that are essential in the assessment of tumor angiogenesis and aggressiveness [4]; common DCE-MRI imaging protocols and analysis methods are an important tool in both preclinical and clinical research [5]. ^18^Fluoro-2- deoxyglucose- positron emission tomography (FDG-PET) assesses intratumoral glucose metabolism with parameters associated with tumor angiogenesis, including the maximum standardized uptake value (SUV_max_) and metabolic tumor volume (MTV), thereby providing biological and physiological information about tumor viability [6–8]. Both PET and MRI bear potential for non-invasive assessment, and hybrid PET/MR imaging may be suitable for precise evaluation. DCE-integratedMR-PET combines the advantages of PET metabolic analysis and MRI permeability imaging for an integral evaluation and thus provides a powerful non-invasive imaging technology to assess tumor biology.

In addition, tumor angiogenesis can also be indirectly evaluated by serum analysis. Serum vascular endothelial growth factor (VEGF)-A, angiogenin, and angiopoietin-1 (Ang-1), which are secreted by tumors in the body and thus indicate total tumor growth and aggressiveness, are considered biomarkers for tumor angiogenesis [9–11].

Given that tumor angiogenesis, an important prognostic indicator in NSCLC, can be evaluated by both imaging and serum analyses [12, 13], we investigated whether radiologic parameters derived from DCE-integratedMR-PET scans correlate with serum angiogenesis-related biomarkers. The present study could provide a non-invasive method for the quantitative assessment of tumor angiogenesis in NSCLC and might guide the use of angiogenesis inhibitors in clinical practice.

## Methods

### Study design and patient enrolment

This prospective study was conducted according to the guidelines of the Declaration of Helsinki and its later amendments, and was approved by the National Taiwan University Hospital Research Ethics Committee (approval number: 201712101RIND). Written informed consent was obtained from all participants after the nature of the procedures had been fully explained.

Patients with a new diagnosis of NSCLC were enrolled, and they underwent DCE-integratedMR-PET scans before treatment and were staged using the American Joint Committee on Cancer staging manual (8th edition) [14]. Histopathological reviews were carried out by an experienced pathologist who majored in thoracic oncology. For patients with adenocarcinoma, cancer specimens were analyzed using RNA reverse transcription-polymerase chain reaction or direct DNA sequencing, as previously described [15]. Epidermal growth factor receptor (EGFR) mutation was defined as the presence of an EGFR exon19del or L858R mutation in tumor genomic DNA, and anaplastic lymphoma kinase (ALK)/c-ros oncogene 1 (ROS1) rearrangement was defined as the occurrence of an ALK or ROS1 rearrangement in tumor genomic DNA. A total of 15 healthy individuals (median age, 55 years; range, 33–70 years; 5 women and 10 men) served as healthy controls.

### Magnetic resonance-positron emission tomography examinations

The simultaneous 3-TMR-PET machine (Biograph mMR; Siemens Healthcare, Erlangen, Germany) was used to perform MR-PET imaging, as previously described [4, 16]. The examination time was approximately one hour. For obtaining positron emission tomography images, intravenous administration of 3.3 MBq of FDG per kg body weight (range, 132–300 MBq) was performed on all patients after they were made to fast; images were obtained at 60 min after injection; a 4-min scan with 864–1335 mm in length was applied for one bed position, to a total of five bed positions with a total acquisition time of 20 min per patient, per standard protocol for whole-body PET images. For image reconstruction, the ordered-subsetexpectation-maximization iterative algorithm with a 5-mmpost-reconstruction Gaussian filter was used.

For obtaining magnetic resonance images, a contrast agent (gadobutrol [0.2 mmol/kg]; Gadavist, Bayer Healthcare, Berlin, Germany) was intravenously administered. Axial volumetric interpolated breath-hold examination sequence was performed with parameters as follows: repetition time [TR]/echo time [TE] 4.03/1.45 ms; slice thickness 2 mm; field of view 320 × 260 mm; fractional anisotropy (FA) 9°; matrix size 320 × 250; number of excitations (NEX) 1; image acquisition time 95 s in five divided scans. The monoexponential function (b-values: 50, 200, 600, and 1000 s/mm^2^) was used to construct ADC maps.

### Magnetic resonance-positron emission tomography image analysis

The two-compartment Tofts model and after-motion registration were employed for pharmacodynamic analyses of magnetic resonance images. Two observers (two radiologists who specialized in chest imaging, with 8 and 22 years of experience in chest MRI, respectively) drew freehand the regions of interest in consensus. Image parameters of the largest primary lung tumor seen on T1-weighted MRI (contrast-enhanced) were evaluated using a commercial software (MIStar; Apollo Medical Imaging Technology, Melbourne, Australia): reverse reflux rate constant (*k*_ep_), volume transfer constant (*K*^trans^), blood plasma volume fraction (*v*_*p*_), extracellular extravascular volume fraction (*v*_*e*_), apparent diffusion coefficient (ADC), and initial area under the time-to-signal intensity curve at 60 s post enhancement (iAUC_60_). ADC indicates the amount of water diffusion within tumors; *K*^trans^ evaluates the diffusive transport of low-molecular-weight gadolinium chelates across the capillary endothelium; and *k*_ep_, *v*_*p*_, and *v*_*e*_, which indicate the reflux rate constant, plasma volume, and extracellular volume, respectively, are regarded as biomarkers predictive of tumor angiogenesis. The MTV was defined as the sum of the volumes of the primary lung tumor and the regional lymphadenopathy using a threshold of 40% of the maximal SUV, assessed through semi-automatically conducted 3-dimensional outlining by the commercial software (Syngo.via; Siemens Healthcare, Erlangen, Germany) [6, 16]. The median total time of image analysis for each set of images was 45 min (range, 30–60 min).

### Quantification of serum angiogenic biomarkers in patients with NSCLC

Serum samples were collected before DCE-integratedMR-PET scans. A 10-mL blood sample was drawn from each patient, placed in vacutainer red-topped tubes (Becton Dickinson and Company, New Jersey, USA), allowed to clot at room temperature for 30 min, and then centrifuged at 1140×*g* (2500 rpm) for 30 min. Serum was aliquoted and stored aseptically at − 80 °C until analysis. Quantitative serum angiogenic biomarker enzyme-linked immunosorbent assay kits (R&D Systems, Minneapolis, USA) were used to measure VEGF-A, Ang-1, and angiogenin concentrations in the serum samples [17].

### Treatments and follow-up

Treatment was performed according to the institutional guideline for NSCLC. The use of surgery, radiotherapy, chemotherapy, immunotherapy, targeted agents, or clinical trial enrolment was recommended by the institutional multidisciplinary lung cancer panel discussion to improve patients’ clinical outcomes. Angiogenesis inhibitors approved by the Taiwan Food and Drug Administration for the treatment of selected NSCLC patients, including bevacizumab and ramucirumab, were administered at the discretion of the attending physician.

Patients underwent chest radiography every 2–4 weeks and computed tomography (CT) of the brain and chest (including the liver and adrenal glands) every 2–3 months as routine clinical practice, and other imaging studies, including PET or MRI, were conducted when necessary. Overall survival (OS) was calculated as the time from the start of any treatment until death from any cause. The median follow-up period was 27 months for surviving patients (range, 2–56 months).

### Statistical analyses

The Statistical Package for Social Sciences for Windows, version 17.0 (SPSS, Chicago, IL, USA) was used for statistical analyses. Statistical comparisons of serum VEGF-A, Ang-1, and angiogenin concentrations between patients and healthy controls were performed using unpaired Student’s t-tests. The correlation between DCE-integratedMR-PET parameters and serum angiogenesis-related biomarkers was evaluated by Pearson’s linear regression analysis. Statistical comparisons of MR-PET-derived parameters between clinical groups (male vs. female, advanced vs. early stage, adenocarcinoma vs. non-adenocarcinoma, EGFR mutation found vs. not found, and ALK/ROS1 rearrangement found vs. not found) were performed using unpaired Student’s t-tests. When Bonferroni correction was applied for multiple comparisons (a total of eight MR-PET derived parameters), *p*-values less than 0.006, a threshold obtained by dividing 0.05 by the number of tests (eight), were considered statistically significant [18]. Survival analyses were conducted using the data available on July 31, 2020. Kaplan–Meier life-table analyses were used to assess survival rates, and log-rank tests were used for prognostic parameter evaluations.

## Results

### Patient characteristics

**Fig. 1:**
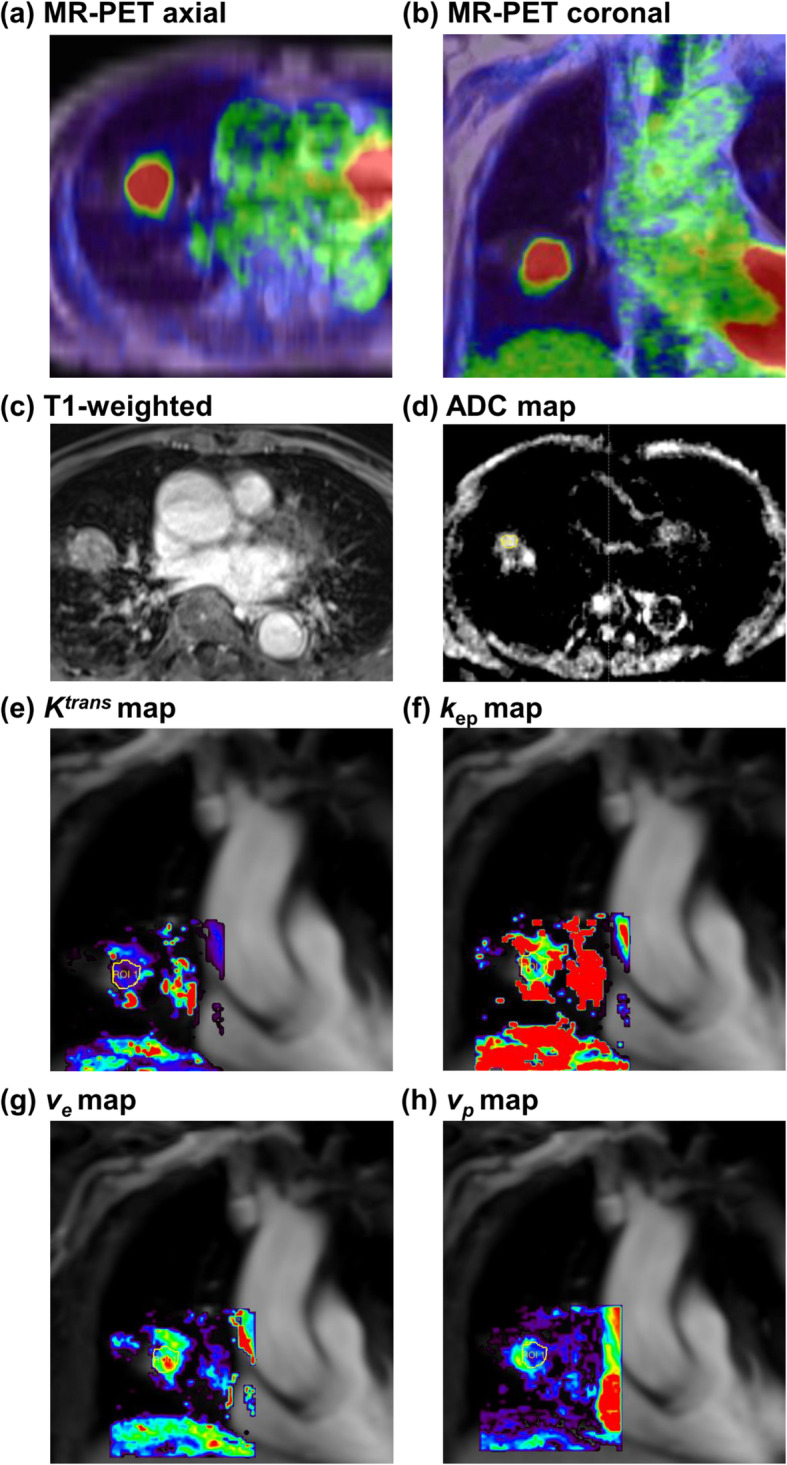
Dynamic contrast-enhanced integrated magnetic resonance-positron emission tomography parameters obtained at diagnosis in a patient with right lower lung adenocarcinoma (cT2aN0M0, stage IB). Measured magnetic resonance-positron emission tomography (MR-PET) parameters were as follows: (**a, b**) SUV_max_, 11; (**c**) tumor size, 3.0 cm (by T1-weighted post contrast); (**d**) ADC, 1025 (10^− 6^ mm^2^/s); (**e**) *K*^trans^, 110 (10^− 3^/min); (**f**) *k*_ep_, 598 (10^− 3^/min); (**g**) *v*_*e*_, 202 (10^− 3^); (**h**) *v*_*p*_, 107 (10^− 3^); and iAUC_60_, 253 (10^− 3^). Measured serum angiogenesis-related biomarkers included VEGF-A (119 pg/mL), angiopoietin-1 (46 ng/mL), angiopoietin-2 (128 pg/mL), and angiogenin (632 ng/mL). Due to medically inoperable status, the patient underwent stereotactic body radiation therapy. The patient achieved a complete response and remained disease-free for 48 months

**Fig. 2:**
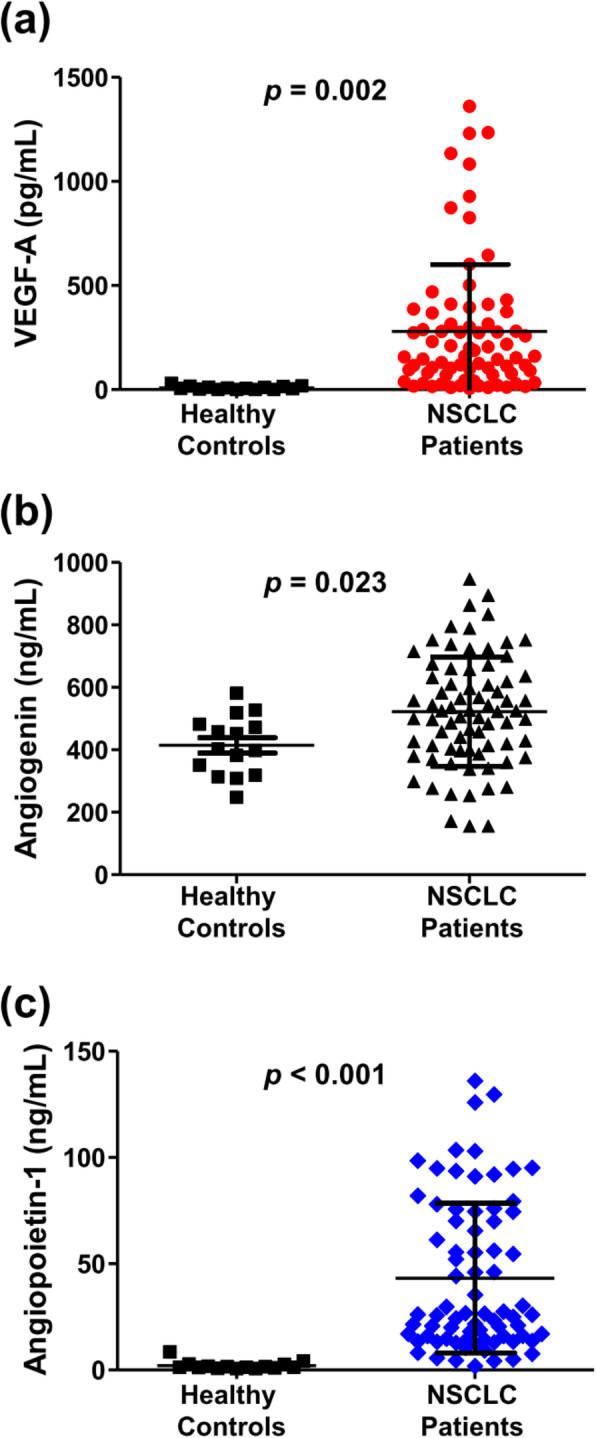
Comparisons of serum VEGF-A, angiogenin, and angiopoietin-1 concentrations between non-small-cell lung carcinoma (NSCLC) patients and healthy controls. Serum (**a**) VEGF-A, (**b**) angiogenin, and (**c**) angiopoietin-1 concentrations in patients and healthy controls. *P-values* for statistical comparisons were obtained using the unpaired Student’s t-test

Between March 2017 and December 2018, 75 patients newly diagnosed with NSCLC (26 women, 49 men; median age: 65 years; range: 40–80 years; Table 1) were prospectively enrolled. A patient’s DCE-integratedMR-PET images are shown in Fig. 1. Fifty-six tumors (75%) were adenocarcinomas, 18 (24%) were squamous cell carcinomas, and one (1%) was a pleomorphic carcinoma. More than one-third of the patients (36%) had stage IV disease at diagnosis. Tumor genomic testing revealed that 28 patients (37%) carried an EGFR mutation, and three (4%) had an ALK/ROS1 rearrangement. Serum VEGF-A (280 pg/mL in NSCLC patients vs. 9 pg/mL in healthy controls; *p* = 0.002), angiogenin (522 ng/mL in NSCLC patients vs. 414 ng/mL in healthy controls; *p* = 0.023), and Ang-1 (mean: 43 ng/mL in NSCLC patients vs. 2 ng/mL in healthy controls; *p* <  0.001) concentrations were significantly elevated in NSCLC patients compared with healthy individuals (Fig. 2).

**Table 1:** Patient clinical characteristics (*n* = 75)

	n	%
Age (years)		
Median (range)	65 (40–80)	
Sex		
Male	49	65
Female	26	35
Stage grouping*		
I	12	16
II	5	7
III	31	41
IV	27	36
Histology		
Adenocarcinoma	56	75
Squamous cell carcinoma	18	24
Pleomorphic carcinoma	1	1
EGFR status^#^		
Mutation not found	47	63
Mutation found	28	37
ALK/ROS1 status^†^		
Rearrangement not found	72	96
Rearrangement found	3	4

*Abbreviations:* EGFR = epidermal growth factor receptor; ALK = anaplastic lymphoma kinase; ROS1 = c-ros oncogene 1

* Staging was performed according to the guidelines of the American Joint Committee on Cancer, 8th edition

^#^ EGFR mutation was defined as the presence of an EGFR exon19del or L858R mutation in tumor genomic DNA

† ALK/ROS1 rearrangement was defined as the presence of an *ALK* or *ROS1* rearrangement in tumor genomic DNA

### Correlations of MR-PET parameters with serum angiogenesis-related biomarkers

**Fig. 3:**
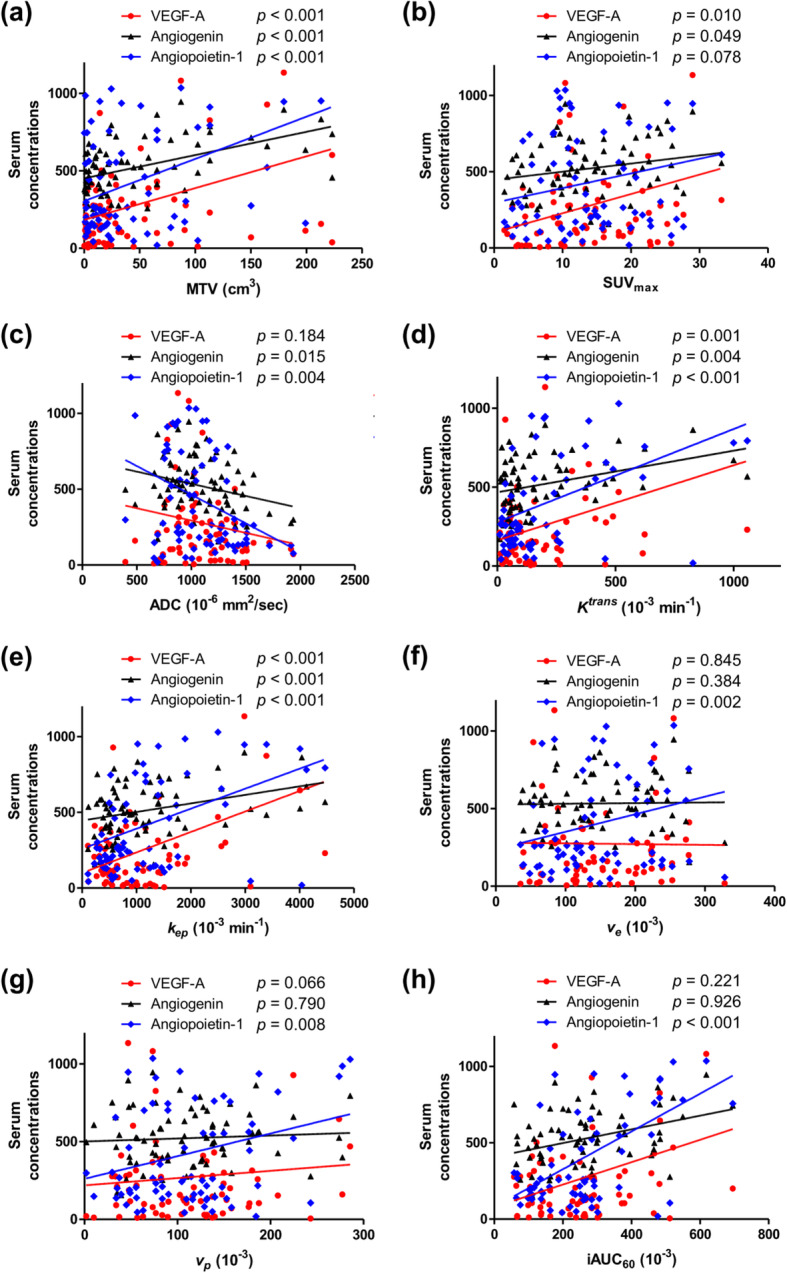
Relationships between MR-PET parameters and serum angiogenesis-related biomarkers in patients with non-small-cell lung carcinoma. Scatterplots demonstrating the relationships between serum VEGF-A (pg/mL), angiogenin (ng/mL), and angiopoietin-1 (10^− 1^ ng/ml) concentrations and (**a**) metabolic tumor volume (MTV), (**b**) SUV_max_, (**c**) ADC, (**d**) *K*^*trans*^, (**e**) *k*_ep_, (**f**) *v*_*e*_, (**g**) *v*_*p*_, and (**h**) iAUC_60_. *P*-values for statistical comparisons between MR-PET parameters and serum angiogenesis-related biomarkers were obtained using Pearson’s linear regression analysis. When Bonferroni correction was applied for multiple comparisons (a total of eight MR-PET derived parameters), *p*-values < 0.006 were considered statistically significant

The associations between eight MR-PET parameters and three serum angiogenesis-related biomarkers were evaluated (Table 2). When Bonferroni correction was applied for multiple comparisons (a total of eight MR-PET derived parameters), *p*-values < 0.006, a threshold obtained by dividing 0.05 by the number of tests (eight), were considered statistically significant. As shown in Fig. 3, three MR-PET measures, namely MTV, *K*^trans^, and *k*_ep_, showed strong linear correlations (*p* <  0.001) with all the tested serum angiogenesis-related biomarkers, i.e., VEGF-A, angiogenin, and Ang-1, indicating that quantification MR-PET measurement reflected the concentrations of serum angiogenesis-related biomarkers. Three other MR-PET measures, i.e., ADC (*p* = 0.004), *v*_*e*_ (*p* = 0.002), and iAUC_60_ (*p* <  0.001), showed significant correlations with Ang-1, one of the tested angiogenesis-related biomarkers.

**Table 2:** Correlation between dynamic contrast-enhanced integrated MR-PET parameters and serum angiogenesis-related biomarkers

	VEGF-A (pg/mL)			Angiogenin (ng/mL)			Angiopoietin-1 (ng/mL)		
	*R*^*2*^	Slope	*p*-value*	*R*^*2*^	Slope	*p*-value*	*R*^*2*^	Slope	*p*-value*
MTV (cm^3^)	0.16	2.26	*< 0.001*^*†*^	0.22	1.45	*< 0.001*^*†*^	0.22	0.29	*< 0.001*^*†*^
SUV_max_	0.09	12.67	*0.010*	0.05	5.34	*0.049*	0.04	0.96	*0.078*
ADC (10^−6^ mm^2^/sec)	0.03	−0.16	*0.184*	0.08	−0.16	*0.015*	0.11	−0.04	*0.004*^*†*^
*K*^*trans*^ (10^−3^ min^−1^)	0.14	0.24	*0.001*^*†*^	0.11	0.07	*0.004*^*†*^	0.26	0.04	*< 0.001*^*†*^
*k*_ep_ (10^−3^ min^− 1^)	0.31	0.13	*< 0.001*^*†*^	0.20	0.06	*< 0.001*^*†*^	0.21	0.01	*< 0.001*^*†*^
*v*_*e*_ (10^−3^)	< 0.01	0.04	*0.845*	0.01	−0.11	*0.384*	0.12	0.07	*0.002*^*†*^
*v*_*p*_ (10^−3^)	0.05	0.81	*0.066*	< 0.01	0.06	*0.790*	0.09	0.13	*0.008*
iAUC_60_ (10^−3^)	0.02	0.13	*0.221*	< 0.01	0.01	*0.926*	0.20	0.05	*< 0.001*^*†*^

*Abbreviations*: MR-PET = magnetic resonance-positron emission tomography; VEGF = vascular endothelial growth factor; MTV = metabolic tumor volume; SUV_max_ = maximum standardized uptake value; *k*_ep_ *=* reverse reflux rate constant; *K*^trans^ *=* volume transfer constant; *v*_*p*_ = blood plasma volume fraction; *v*_*e*_ = extracellular extravascular volume fraction; ADC = apparent diffusion coefficient; iAUC_60_ = initial area under the time-to-signal intensity curve at 60 s post enhancement

* Significance tested using Pearson’s linear regression analysis

^†^ When Bonferroni correction was applied for multiple comparisons (a total of eight MR-PET derived parameters), *p*-values < 0.006 were considered statistically significant

### MR-PET parameters and serum angiogenesis-related biomarkers in patients with clinically advanced disease

**Fig. 4:**
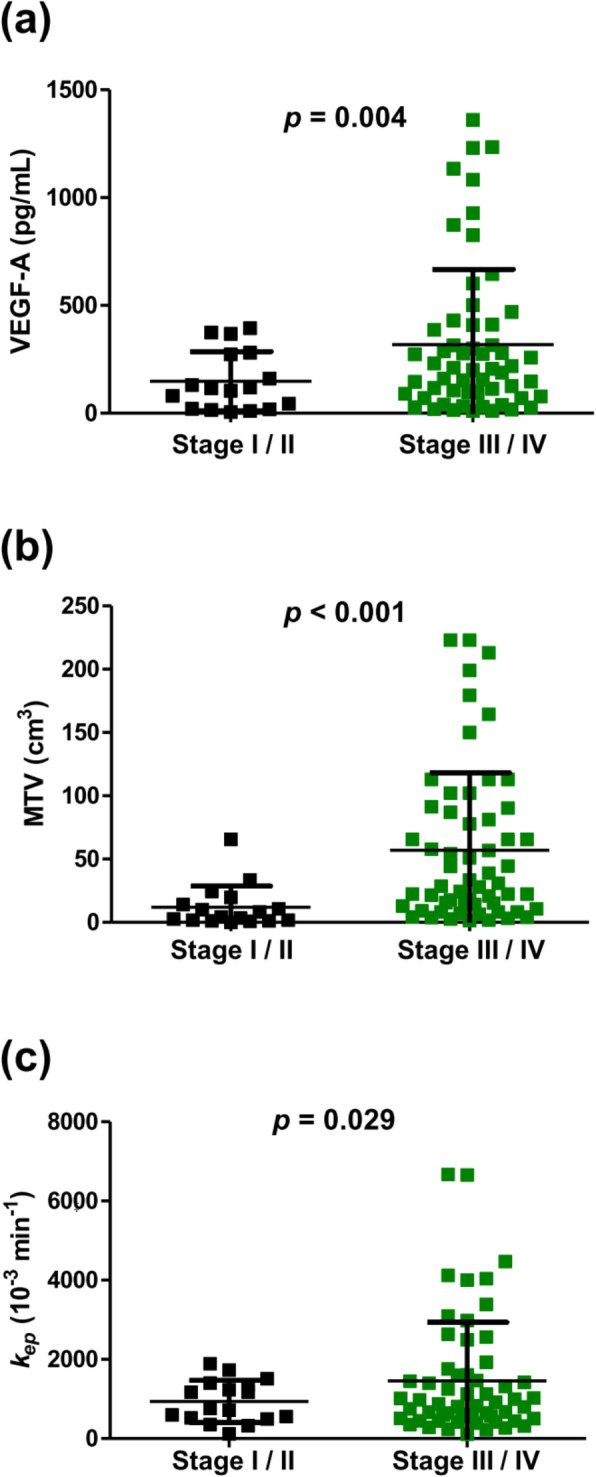
MR-PET parameters and serum angiogenesis-related biomarkers in early- and advanced-stage non-small-cell lung carcinoma patients. Comparisons of (**a**) serum VEGF-A (pg/mL), (**b**) MR-PET derived-maximum metabolic tumor volume (MTV), and (**c**) MR-PET derived- *k*_ep_ between early- and advanced-stage non-small-cell lung carcinoma patients. *P-values* for statistical comparisons were obtained using the unpaired Student’s t-test

We evaluated the association of MR-PET measures and serum biomarkers with clinical tumor characteristics (Table 3). As shown in Fig. 4, serum VEGF-A concentrations (318 ± 349 vs. 148 ± 138 pg/mL, *p* = 0.004), MTV values (57 ± 61 vs. 12 ± 17 cm^3^, *p* <  0.001), and *k*_ep_ values (1455 ± 779 vs. 935 ± 532 10^− 3^/min, *p* = 0.029) were significantly higher in patients with advanced disease (stage III or IV) than in those with early-stage disease (stage I or II). No significant correlations were found between MR-PET or serum measures and other clinical characteristics, including sex, histology, EGFR mutation, or ALK/ROS1 rearrangement.

**Table 3:** Correlations between angiogenesis-related biomarkers and clinical tumor characteristics (n = 75)

(mean ± standard deviation) *p-value*^※^	Sex		Tumor stage*		Histology		EGFR Mutation^#^		ALK/ROS1 Rearrangement^†^	
	Male	Female	Stage I / II	Stage III / IV	Adenocarcinoma	Non-Adenocarcinoma	Found	Not found	Found	Not found
VEGF-A (pg/mL)	326 ± 353	192 ± 231	148 ± 138	318 ± 349	268 ± 309	313 ± 361	254 ± 317	293 ± 326	389 ± 601	275 ± 311
	*0.053*		*0.004*^*‡*^		*0.632*		*0.621*		*0.774*	
Angiogenin (ng/mL)	539 ± 172	491 ± 180	473 ± 111	537 ± 188	504 ± 178	577 ± 160	430 ± 161	571 ± 1 64	735 ± 203	513 ± 170
	*0.174*		*0.091*		*0.102*		*0.061*		*0.196*	
Angiopoietin-1 (ng/mL)	49 ± 39	32 ± 23	43 ± 31	44 ± 37	38 ± 31	57 ± 44	35 ± 30	47 ± 37	53 ± 44	43 ± 35
	*0.055*		*0.931*		*0.095*		*0.139*		*0.739*	
MTV (cm^3^)	56 ± 61	30 ± 46	12 ± 17	57 ± 61	30 ± 38	96 ± 74	32 ± 37	60 ± 62	82 ± 71	45 ± 57
	*0.065*		*< 0.001*^*‡*^		*0.108*		*0.139*		*0.469*	
SUV_max_	15 ± 8	12 ± 6	9 ± 6	15 ± 7	13 ± 7	18 ± 8	12 ± 7	15 ± 7	10 ± 6	14 ± 8
	*0.070*		*0.101*		*0.112*		*0.156*		*0.355*	
ADC (10^− 6^ mm^2^/sec)	1056 ± 296	1137 ± 324	1145 ± 499	876 ± 236	1122 ± 321	975 ± 231	1077 ± 321	1088 ± 301	1064 ± 375	1085 ± 306
	*0.293*		*0.190*		*0.059*		*0.883*		*0.931*	
*K*^*trans*^ (10^− 3^ min^− 1^)	392 ± 556	197 ± 333	283 ± 430	464 ± 675	332 ± 556	302 ± 265	401 ± 676	284 ± 371	633 ± 1011	312 ± 474
	*0.063*		*0.310*		*0.754*		*0.420*		*0.638*	
*k*_ep_ (10^− 3^ min^− 1^)	1484 ± 1350	1062 ± 1300	935 ± 532	1455 ± 779	1211 ± 1337	1710 ± 1310	1066 ± 931	1482 ± 1501	2689 ± 3436	1281 ± 1207
	*0.193*		*0.029*^*‡*^		*0.163*		*0.144*		*0.552*	
*v*_*e*_ (10^− 3^)	206 ± 192	144 ± 83	286 ± 280	155 ± 97	188 ± 185	176 ± 85	239 ± 253	156 ± 79	135 ± 111	187 ± 167
	*0.052*		*0.173*		*0.722*		*0.115*		*0.513*	
*v*_*p*_ (10^− 3^)	121 ± 99	117 ± 50	114 ± 74	121 ± 88	119 ± 92	120 ± 60	139 ± 118	109 ± 59	75 ± 39	121 ± 86
	*0.830*		*0.729*		*0.984*		*0.242*		*0.159*	
iAUC_60_ (10^− 3^)	378 ± 412	266 ± 135	511 ± 622	289 ± 187	350 ± 393	307 ± 127	458 ± 536	276 ± 150	300 ± 285	341 ± 349
	*0.086*		*0.165*		*0.471*		*0.102*		*0.831*	

*Abbreviations*: MTV = metabolic tumor volume; SUV_max_ = maximum standardized uptake value; *k*_ep_ *=* reverse reflux rate constant; *K*^trans^ *=* volume transfer constant; *v*_*p*_ = blood plasma volume fraction; *v*_*e*_ = extracellular extravascular volume fraction; ADC = apparent diffusion coefficient; iAUC_60_ = initial area under the time-to-signal intensity curve at 60 s post enhancement

* Tumor stage was classified by the American Joint Committee on Cancer 8th edition

^#^ EGFR mutation was defined as the presence of an EGFR exon19del or L858R mutation in tumor genomic DNA

† Anaplastic lymphoma kinase (ALK)/c-ros oncogene 1 (ROS1) rearrangement was defined as the presence of an *ALK* or *ROS1* rearrangement in tumor genomic DNA

^※^Significance was tested using Student’s t-test

^‡^ *P-values* < 0.05 were considered statistically significant

### Potential of MR-PET parameters in predicting the efficacy of angiogenesis inhibitors

**Fig. 5:**
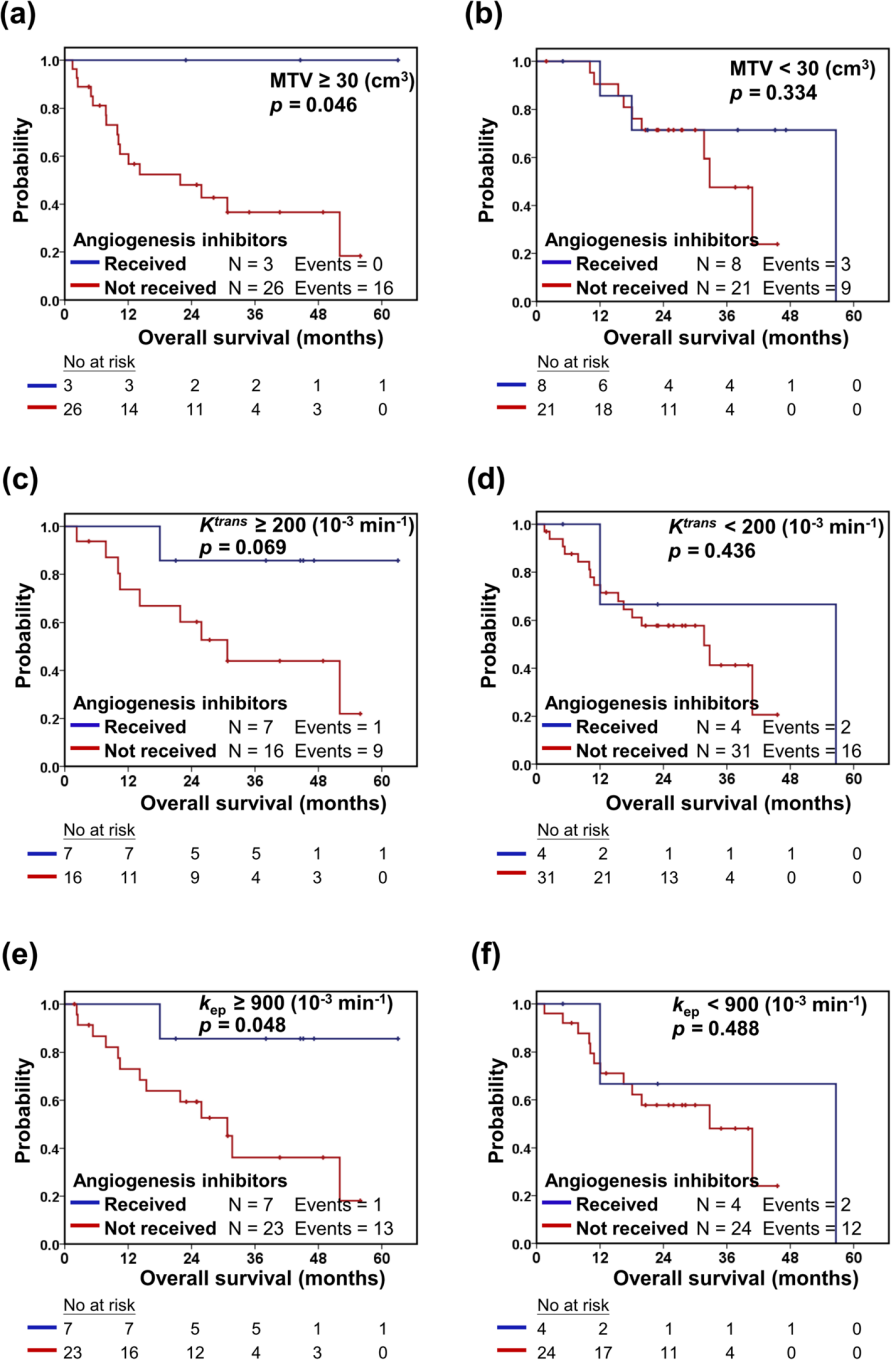
Overall survival analysis in advanced-stage patients who received or did not receive angiogenesis inhibitors. Patients were grouped according to initial angiogenesis-related MR-PET parameters, i.e., (**a**) metabolic tumor volume (MTV) ≥ 30 or (**b**) < 30 cm^3^, (**c**) *K*^trans^ ≥ 200 or (**d**) < 200 (10^− 3^ min^− 1^), and (**e**) *k*_ep_ ≥ 900 or (**f**) < 900 (10^− 3^ min^− 1^). P-values were determined using Kaplan–Meier log-rank tests

During the median follow-up period of 27 months (range, 2–56 months), 11 of the 58 patients with advanced disease (stage III or IV) received angiogenesis inhibitors (bevacizumab or ramucirumab) as part of their treatment. The survival of advanced-stage patients, grouped by initial angiogenesis-relatedMR-PET parameters, was investigated (Table 4). The grouping was based on median values of MTV, *K*^trans^, or *k*_ep_ in patients with advanced disease. In advanced-stage patients with initial higher angiogenesis-relatedMR-PET parameters (Fig. 5), including MTV > 30 cm^3^ (*p* = 0.046), *K*^trans^ > 200 10^− 3^/min (*p* = 0.069), and *k*_ep_ > 900 10^− 3^/min (*p* = 0.048), a significantly longer OS was seen when angiogenesis inhibitors were administered. However, no significant survival difference was found when angiogenesis inhibitors were administered in patients with lower initial MTV, *K*^trans^, or *k*_ep_.

**Table 4:** Overall survival analysis in advanced-stage patients who received or did not receive angiogenesis inhibitors

	2-year overall survival (%)		*p-*value*
	Angiogenesis inhibitors received (*n* = 11)	Angiogenesis inhibitors not received (*n* = 47)	
MTV (cm^3^)			
≥ 30	100.0	48.1	*0.046*^*‡*^
< 30	71.4	71.4	*0.334*
*K*^*trans*^ (10^−3^ min^−1^)			
≥ 200	85.7	60.3	*0.069*
< 200	66.7	57.8	*0.436*
*k*_ep_ (10^−3^ min^−1^)			
≥ 900	85.7	59.3	*0.048*^*‡*^
< 900	66.7	57.8	*0.488*

*Abbreviations*: MR-PET = magnetic resonance positron emission tomography; MTV = metabolic tumor volume; *K*^trans^ *=* volume transfer constant; *k*_ep_ *=* reverse reflux rate constant

* Significance was tested using Kaplan–Meier analysis and log-rank tests

^‡^ *p*-values < 0.05 were considered statistically significant

## Discussion

This is the first study, to our knowledge, to investigate correlations between DCE-integratedMR-PET imaging and serum biomarkers quantitatively assessing tumor angiogenesis in NSCLC patients. Importantly, we found that MTV, *K*^trans^, and *k*_ep_ values showed significant and positive correlations with serum angiogenesis-related biomarkers, and radiologic and serum biomarkers were associated with advanced tumor stage. Our findings indicate that patients with higher initial angiogenesis-relatedMR-PET parameters may have benefited from angiogenesis inhibitors, and the imaging biomarkers could be potentially used to guide the clinical use of angiogenesis inhibitors. This study provides a non-invasive, reliable method for the quantitative assessment of tumor angiogenesis in NSCLC patients.

Novel imaging parameters have been demonstrated to be useful in the evaluation of tumor angiogenesis in NSCLC [19]. The ADC value in DW-MRI, indicating the amount of water diffusion within tumors, has been shown to be inversely correlated with tumor angiogenesis, while the parameters MTV and SUV_max_ in FDG-PET studies correlate with tumor aggressiveness [7, 20]. In our study, image parameters showed significant positive correlations with the concentration of serum angiogenesis biomarkers, further supporting the application of MR-PET parameters in tumor evaluation [21, 22]. In addition, accumulating studies have examined imaging parameters and serum angiogenesis-related biomarkers in several solid tumors, including NSCLC, rectal cancer, prostate cancer, and breast cancer. For example, Kaira et al. included 37 NSCLC patients and demonstrated positive correlations between IHC VEGF and ^18^F-FDG/PET-derived SUV_max_ [8]. In patients with rectal cancer, George et al. studied 31 patients and found a positive correlation between serum VEGF and MR-derived*K*^trans^ [23], Atkin et al. evaluated 15 patients and demonstrated a positive correlation between serum VEGF and IHC microvascular density (MVD) [24], and Yeo et al. analyzed 46 patients and demonstrated a positive correlation between IHC MVD and MR-derived*k*_ep_ [25]. In patients with prostate cancer, Oto et al. evaluated 73 men and found a negative correlation between the Gleason score and MR-derived ADC and a positive correlation between IHC MVD and *v*_*e*_ [26]. In patients with breast cancer, Kim et al. studied 81 women and found a positive correlation between IHC MVD and *v*_*e*_ [27]. These previous findings, which are in line with our results, demonstrate that MR-PET imaging and serum biomarkers can be used to quantitatively assess tumor angiogenesis.

In our study, higher serum and radiologic biomarkers were detected in non-adenocarcinoma tumors than in adenocarcinomas, although the differences were not statistically significant. These findings can be partly explained by the higher proportion of advanced-stage (stage III or IV) tumors in the non-adenocarcinoma group than in the adenocarcinoma group (89% vs. 73%, *p* <  0.001). Consistently, previous studies also showed that non-adenocarcinoma tumors had higher SUV and Ki67 scores and were characterized by higher tumor aggressiveness [28, 29]. Whether non-adenocaricnoma tumors poccessed with dissimilar proliferation pattern, glucose metabolism, or aggressiveness than adenocarcinoma tumors need further investigation.

Our study showed that patients with higher initial angiogenesis-relatedMR-PET parameters (including MTV > 30 cm^3^, *K*^trans^ > 200 10^− 3^/min, and *k*_ep_ > 900 10^− 3^/min) may have benefited from angiogenesis inhibitors, resulting in longer survival. Consistently, de Langen et al. demonstrated that patients with a significant decrease in SUV or tumor perfusion three weeks post bevacizumab and erlotinib treatment had longer survival [12], and Kelly et al. demonstrated correlations between changes in *k*_ep_ and serum basic fibroblast growth factor and progression-free survival in patients who received sorafenib [13]. The above-mentioned findings, in line with our results, further demonstrate radiological and cytokine changes as biomarkers indicative of early angiogenesis inhibition, and these biomarkers can be used to identify patients who may benefit from angiogenesis inhibitors.

Novel PET radiopharmaceuticals for imaging angiogenesis are under investigation in lung cancer patients, and α_v_β_3_ integrin, which upregulates activated neovascular endothelial cells in association with tumor angiogenesis, has been targeted for PET imaging [30]. Arg-Gly-Asp (RGD) peptide-based PET tracers, which have been developed to image integrin expression in tumors and are predominantly used in preclinical environment with clinical implementation, are being studied at present [31, 32]. Since the correlation of endothelial integrin and glucose metabolism in malignant lesions needs further assessment, preliminary results from novel PET radiopharmaceuticals warrant attentive interpretation for response evaluation for targeted molecular therapies with antiangiogenic or integrin-targeted agents.

Our study has several limitations. A total of 75 NSCLC patients and 15 healthy controls were included in the study; the number of patients and controls are not balanced. However, since the study was not a case-control study or paired comparison analysis, and unpaired Student’s t-tests were performed for statistical analysis between patients and controls, the imbalance does not influence the accuracy of our data interpretation. Serum angiogenesis biomarkers are thought to represent the average concentrations secreted by all tumors in the body, including the main tumor; thus, the serum biomarker concentrations might indicate integral tumor aggressiveness but might not comprehensively reflect individual tumor heterogeneity.

DCE-integrated MR-PET imaging presents as being a promising non-invasive method for assessing tumor angiogenesis in patients with NSCLC. Our findings suggest that DCE-integratedMR-PET imaging may be useful for assessing angiogenesis in patients with NSCLC at diagnosis, identifying patients who may benefit from being treated with angiogenesis inhibitors, and monitoring the response to therapies.

## Conclusions

Radiologic parameters derived from DCE-integratedMR-PET scans correlated with serum angiogenesis-related biomarkers in NSCLC patients and could be used to potentially guide the clinical use of angiogenesis inhibitors. Since tumor angiogenesis is an important prognostic factor for anticancer treatment and patient survival, our results suggest that DCE-integratedMR-PET imaging, which provides a non-invasive, quantitative assessment of tumor angiogenesis, may play a role in personalized medicine for patients with NSCLC.

## Data Availability

The datasets used and/or analyzed during the current study are available from the corresponding author on reasonable request.

## Abbreviations

NSCLC: non-small-cell lung cancer
DCE: dynamic contrast-enhanced
MRI: magnetic resonance imaging
*K*^trans^: volume transfer constant
*k*_ep_: reverse reflux rate constant
*v*_*e*_: extracellular extravascular volume fraction
*v*_*p*_: blood plasma volume fraction
ADC: apparent diffusion coefficient
iAUC_60_: initial area under the time-to-signal intensity curve at 60 s post enhancement
FDG-PET: ^18^Fluoro-2-deoxyglucose-positron emission tomography
SUV_max_: maximum standardized uptake value
MTV: metabolic tumor volume
VEGF: vascular endothelial growth factor
EGFR: epidermal growth factor receptor
ALK: anaplastic lymphoma kinase
ROS1: c-ros oncogene 1
TR: repetition time
TE: echo time
FA: fractional anisotropy
NEX: number of excitations
DW: diffusion-weighted
CT: computed tomography
OS: overall survival
MVD: microvascular density
